# Examining Associations between Community Health Worker-Rated Health and Mental Health among Latino Adults with Chronic Disease

**DOI:** 10.3390/ijerph18010100

**Published:** 2020-12-25

**Authors:** Kiera Coulter, Maia Ingram, Abby M. Lohr, Melanie L. Bell, Scott Carvajal

**Affiliations:** 1 Department of Health Promotion Sciences, University of Arizona, Tucson, AZ 85724, USA; maiai@arizona.edu (M.I.); abbylohr@arizona.edu (A.M.L.); carvajal@arizona.edu (S.C.); 2 Department of Epidemiology & Biostatistics, University of Arizona, Tucson, AZ 85724, USA; melaniebell@arizona.edu

**Keywords:** community health workers, CHW-rated health, Latinos, mental health

## Abstract

Latinos with chronic disease often experience comorbid depression, but confront barriers to mental health treatment. Community health workers (CHWs) develop trusting relationships with the communities they serve, and may be uniquely positioned to identify Latinos with mental health care needs. Research has not examined whether their rating of clients’ health is indicative of their mental health. This mixed-methods study examines CHWs’ appraisals of Latino adults’ health and their relation to mental health outcomes, and explores factors informing CHWs’ rating of health status. The current study utilized baseline data from the Linking Individual Needs to Community and Clinical Services (LINKS) study. We assessed associations between CHW-rated health (CHWRH), or rating of health status as poor–excellent, and mental health outcomes with multilevel linear regression modelling. We qualitatively analyzed CHWs’ written perceptions of participants’ health status to understand what influenced their health rating. The quantitative results showed that CWHRH was significantly related to depressive symptoms and emotional problems severity. The qualitative results showed that CHWs took a holistic and ecological approach in rating health. The findings suggest that CHWRH could be indicative of mental health among Latino adults. Further studies investigating CHWRH as an independent indicator of mental health are warranted.

## 1. Introduction

Latinos represent the largest and fastest growing racial/ethnic group in the United States (U.S.), accounting for 17% of the U.S. population and are projected to reach 119 million by 2060 [1]. It is imperative to address health disparities confronted by Latinos, who experience a disproportionate burden of chronic diseases and some co-morbid mental health problems. Latinos have the highest lifetime risk for type II diabetes, and are nearly twice as likely relative to non-Latino Whites to be diagnosed with diabetes [2,3]. Key risk factors for cardiovascular disease (i.e., obesity, hypertension, and hypercholesterolemia) have also become increasingly prevalent among Latinos [4]. Chronic disease is also related to Latino mental health given that persons with chronic disease report more mental health problems [5]. Comorbid depression and diabetes has been identified as a risk factor for increased mortality among older Mexican-Americans specifically [6], and has been estimated to be as high as 33.5% in Latino primary care samples [7]. Depression also exacerbates challenges with successful chronic disease management and self-care. Individuals with this comorbidity often need to address simultaneous physical and emotional symptomology (depressed mood, pain, anxiety, etc.) through pharmacologic and non-pharmacologic therapies, while also working to sustain provider-recommended health behaviors (e.g., diet, exercise, and medication adherence) [8,9,10,11].

Given how depression complicates disease management, it is critical that Latinos’ needs and risks for mental health conditions are assessed along with physical conditions (such as chronic disease). However, Latinos face several challenges that impede mental health diagnosis, and in turn contribute to significant treatment disparities [12]. Only 36% of Latinos with depression receive treatment as opposed to 60% of non-Latino Whites [13]. Latinos most commonly receive a depression diagnosis through their primary care provider (PCP), but linguistic and cultural incongruence between Latino patients and PCPs often complicate depression detection [14]. Language proficiency is critical in psychiatric care because an accurate diagnosis depends on communication between patients and physicians. However, studies have shown that lack of English proficiency or culturally competent mental health services prevent Latinos from receiving or utilizing psychiatric care [15,16]. Additionally, researchers have documented that Latinos are more likely than non-Latino Whites to express depression somatically (e.g., aches and pains) [17,18]. Consequentially, health care providers may overlook symptoms of depression or mental health distress for physical health issues [19], particularly in patients with chronic disease given the overlap in somatic symptoms for depression and medical illness [20]. Moreover, cultural stigma regarding mental health, low health literacy, lack of insurance, and misconceptions about mental health treatment are also significant barriers deterring Latinos from receiving timely depression care [21]. These barriers underscore the need for culturally competent health professionals to improve depression detection and service utilization among Latinos, an area where the community health worker (CHW) model is promising.

CHWs are frontline public health workers who are trusted members of and/or have an unusually close understanding of the community served [22]. CHWs have demonstrated success in improving health outcomes among historically disenfranchised patient populations (including Latinos) [23,24,25], and have been increasingly mobilized in mental health interventions in response to deficiencies in linguistically and culturally competent mental health care providers [26]. Because CHWs share a common culture, language, and community with their patients, CHWs are uniquely positioned to address the cultural, social, and economic barriers that often marginalize them from mental health care. Additionally, CHWs develop trusting relationships with patients, which could make them effective in facilitating communication around their mental health. Further research is needed to empirically examine CHWs’ capacity to identify mental health issues among Latino patients specifically, which this study seeks to address.

Given that CHWs are taking a more active role in addressing patients’ mental health and are highly successful in engendering patient rapport, it is critical to assess whether they can contribute to the identification of mental health problems and what other factors may inform CHWs’ rating of patients’ health status. The objectives of the current study are to (1) examine CHWs’ abilities to appraise mental health needs among Latinos by quantitatively analyzing the association between CHW-rated health (CHWRH) and depressive symptomology as well as emotional problems severity and (2) qualitatively analyze what factors influence CHWRH. CHWRH is conceptually related to self-rated health (SRH), which is patients’ own subjective rating of their overall health as excellent, very good, good, fair, or poor. SRH has shown robust associations with patients’ physical and mental health [27,28,29], and has also been studied relative to physician-rated health [30,31]. Among Latinos specifically, fair/poor SRH has been found to be predictive of short-term mortality (2 year mortality risk) among less acculturated Latinos, and predictive of long-term mortality risk among more acculturated Latinos [32]. However, to our knowledge, community health worker-assessed overall patient health has yet to be examined in research, and its potential usefulness in the identification of mental health problems in clinical settings, such as community-based primary care systems, is not known.

## 2. Materials and Methods

### 2.1. Study Setting and Design

Data for the current study were derived from baseline assessment data of the Linking Individual Needs to Community and Clinical Services (LINKS) study, assessing a community-clinical intervention to improve chronic disease risk and emotional well-being among Latinos in three predominantly Mexican-origin communities. Full details on study protocols are published elsewhere [33]. To complete the baseline assessment questions, participants met with a community-based CHW at each of the three sites who facilitated the assessment in English or Spanish. The three were Latina women, who are fully bilingual in English and Spanish. The CHWs were experienced health professionals with extensive training in CHW core competencies but as part of the intervention they received mental health training. Arizona Community Health Workers Association (AzCHOW) staff trained the LINKS CHWs using the following curricula: Behavioral Health Training for Community Health Workers in Primary Care [34], Sonrisa (a curriculum for promoters on addressing depression among Hispanic clients as part of diabetes prevention or management) [19], and Mental Health First Aid [35]. After meeting individually with each CHW to assess their strengths and opportunities for growth, AzCHOW staff conducted monthly group trainings. During the baseline assessment, CHWs also rated participants’ health status and responded to an open-ended question providing their rationale for the health rating based on their interaction with the participant. Thus, the current study utilized a mixed-method design where qualitative data were analyzed to expand upon the quantitative results [36].

### 2.2. Study Population

A total of 189 participants, residents of the Arizona border region, entered the LINKS study and completed the baseline assessment. Eligibility criteria for this study included being at least 21 years of age, not being pregnant, not having a serious mental illness, and having a diagnosed chronic disease or a pre-chronic disease (e.g., glucose intolerance, pre-diabetes, hypertension, and/or high cholesterol), and being able to speak English or Spanish.

### 2.3. Quantitative Measures

Quantitative data were extracted from measures included within the baseline assessment battery. Participants’ depression symptomology was measured from the 10-item Center for Epidemiologic Studies Depression Scale Revised (CES-D-10) [37], which has been found to be reliable among Mexican immigrant samples [38]. Participants indicated the frequency of a depressive symptoms over the past week on a 4-point Likert scale (0 = rarely or some of the time to 3 = all of the time). Participants’ responses were summed to generate a score of 0–30, with higher scores indicating more severe depressive symptomology. Emotional problems severity was measured by the mental health item of the Short Form-8 (SF-8) [39]. Participants rated how much they had been bothered by emotional problems (i.e., irritability or anxiety) over the past month using a 5-point Likert scale ranging from (1 = not at all to 5 = could not do daily activities). SRH was measured from participants’ rating of their own current health status, using the original Behavioral Risk Factor Surveillance System (BRFSS) item [40]. Participants rated their current health status on a 5-point Likert scale (1 = excellent, 2 = very good, 3 = good, 4 = fair, or 5 = poor). CHWRH was measured from CHWs’ rating of participants’ overall health status. CHWs’ rated participants’ health status using one item modified from the BRFSS question to assess CHWs’ perceptions of participants’ health on a 5-point Likert scale (1 = excellent, 2 = very good, 3 = good, 4 = fair, or 5 = poor). Sociodemographic covariates were age, sex, years of education, and nativity (born in the United States or outside of the United States).

### 2.4. Qualitative Data

Qualitative data were derived from CHWs’ written responses to an open-ended question within the baseline assessment. After rating participants’ health, the survey asked CHWs to describe what they discussed during their meeting with the participant that informed their health rating. The survey asked the question: “Please describe your reasons for rating the participant’s health as you did.” CHWs generally wrote 3–10 sentences in English or Spanish.

### 2.5. Analysis

#### 2.5.1. Quantitative

Multilevel linear regression analyses were conducted to examine the associations between CHWRH and participants’ depression symptomology (Model 1) and emotional problems severity (Model 2), while accounting for clustering by assigned-CHW (random factor). Model 1 and Model 2 additionally controlled for SRH and participant demographics (age, sex, years of education, nativity). All continuous predictors (CHWRH, SRH, age, years of education) were grand mean centered. Model 1 and Model 2 were analyzed with a random intercept and random effect of CHWRH, and then only with a random intercept. Changes in models’ likelihood ratio were tested using likelihood ratios tests and used to evaluate the best fitting model. The models with and without CHWRH were also evaluated. When examining models with different random effects, we used restricted maximum likelihood estimation; and when examining models with different fixed effects, we used maximum likelihood estimation [41]. Assumptions of hierarchical linear modeling (normal distribution of dependent variables, homoscedasticity, normal distribution of residuals, and multicollinearity) were examined. A sensitivity analysis using multiple imputation with chained equations (MICE) was conducted using 20 imputations to examine the influence of missing data on the results of Model 1, as there was approximately 9% missingness in the outcome variable. The imputation model included the outcome variable and covariates specified in Model 1, as well as auxiliary covariates found to be associated with the outcome variable (Pearson correlation coefficient > 0.40). All statistical analyses were conducted using R [42].

#### 2.5.2. Qualitative

Two researchers independently reviewed the data and generated potential codes based on the content of CHWs’ written responses. The two researchers then met with a third researcher to review the themes and develop a codebook, which included both themes and subthemes. The two researchers then used the codebook to independently code all of the data in NVivo 12 [43]. The interrater reliability demonstrated at least 80% agreement for all codes. To better understand what influenced CHWs’ rating of health status, the researchers then compared the qualitative data between participants with good CHWRH scores (CHWRH of excellent, very good, or good) and poor CHWRH scores (CHWRH of fair or poor). By comparing the qualitative data across participants with good CHWRH versus poor CHWRH, the researchers could discern what attributes characterized good health status and poor health status from the CHWs’ perspectives.

## 3. Results

Table 1 summarizes the demographic, emotional well-being, SRH, and CHWRH of LINKS participants at baseline. Almost all participants identified as Hispanic or Latino (94.7%). The majority of participants were female (86%), with a mean age of 56.6 years (*SD* = 13.9). The most prevalent chronic disease reported by participants were hypertension (40.2%), diabetes (38.6%), and vision problems (29.9%). The median CES-D-10 score was 5 (IQR = 7). Most participants reported experiencing *slight* to *extreme* emotional problems, and the remaining 39.6% reported not being bothered by emotional problems over the past week. Participants most frequently rated their health as good (40.4%), followed by fair (37.2%), very good (10.1%), poor (9.0%), and excellent (3.2%). CHWs also mostly rated participants’ health as good (36.9%), followed by fair (32.1%), very good (23.0%), poor (4.8%), and excellent (3.2%).

### 3.1. Quantitative

CHWRH and SRH were moderately correlated (r = 0.51). Table 2 displays the results of Model 1 and Model 2. The results of the LRTs confirmed that the random intercept only models provided better fit for Model 1 and Model 2. Additionally, for both Model 1 and Model 2, the model comparisons conducted between the final random intercept models with and without CHWRH showed that the models with CHWRH included better fit the data (Model 1, ∆X^2^(1) = 17.84, *p* = < 0.001; Model 2, ∆X^2^(1) = 10.92, *p* = < 0.001).

Regarding Model 1, CHWRH had a positive and significant association with participants’ depression symptoms (b = 2.52; 95% CI = 1.39, 3.64), where worse CHWRH was associated with higher levels of depressive symptomology. Additional demographic covariates and SRH were not statistically significant.

Regarding Model 2, CHWRH had a positive and significant association with emotional problems severity (b = 0.34, 95% CI = 0.15, 0.54), where worse CHWRH was also associated with higher levels of emotional problems severity. Within Model 2, gender was statistically significant where being female was associated with increased emotional problems severity (b = 0.47; 95% CI = 0.03, 0.90). The remaining demographic covariates and SRH were not significantly associated with emotional problems severity.

The sensitivity analyses for Model 1 yielded similar results. CHWRH again showed a positive and significant association with depression symptoms (b = 2.62; *p* < 0.001). Years of education was also statistically significant after imputation (b = −0.23, *p* = 0.03). SRH and the remaining demographic covariates were not statistically significant.

### 3.2. Qualitative

The objective of the qualitative analysis was to examine contrasts in themes across good and poor CHWRH to elucidate what informed CHWs’ health rating. Table 3 presents the emerging themes and sample quotes from the participants. The breadth of themes evidences CHWs’ insights into participants’ lived experiences and their nuanced approach in rating health status. CHWs emphasized not only physical health and disease self-management, but also emotional well-being and broader social determinants of health (i.e., family stress, financial stress, and social support) as all relevant to their health rating.

When reviewing the qualitative data across good and poor CHWRH, key contrasts in themes emerged. Overall, physical health issues and chronic disease were common to patients with good and poor CHWRH, however, CHWs described patients with good CHWRH as more effective in disease self-management. Those with good CHWRH were noted to have chronic diseases that were “under control,” through exercise (walking, biking, Zumba), losing weight, better nutrition, adhering to medication, and going to regular check-ups.

*“…she says that every day she does exercise and apart from [that] she walks a lot…she tries to do it very early so that the heat does not affect her she also [takes] Zumba classes which [she enjoys] a lot and [has] been able to keep her diabetes and hypertension under control…”*

(CHWRH Good).

Conversely, among patients with a poor CHWRH, CHWs noted unsuccessful disease self-management. CHWs documented patients not sustaining healthy lifestyle behaviors (poor diet, lack of exercise, smoking) nor adhering to medication, which sometimes resulted in severe disease complications.

*“She has diabetes and she doesn’t care too much, [does] not eat healthy and does nothing [for] exercise…”*

(CHWRH Fair).

A second pattern emerged where accounts of physical pain differed along good and poor CHWRH. Among those with good CHWRH, CHWs described more mild pain or pain that was being effectively managed through medication or other treatment. In participants with poor CHWRH, CHWs noted more severe pain from fibromyalgia, arthritis, and trauma (i.e., car accidents) that impeded participants’ daily functioning.

*“Participant says she experienced this pain for 4 years. She says that she worked as a caregiver and had 3 jobs a day caring for very heavy people, this wore out her bones from the waist down and she describes that the pain is a thousand times stronger than giving birth, her doctors told her that there isn’t a cure for her disease and a day will come where she’s going to want to die because she can’t bare the pain...”*

(CHWRH Fair).

Not surprisingly, CHWs were more likely to document CHWRH as poor when they heard participants describe depressive symptoms, anxiety, and stress (including suicidality) among participants. CHWs attributed participants’ depression, anxiety, and stress to a mental health diagnosis and/or broader life issues (family, money, relationships, health).

*“…She told me that she stresses a lot because her husband has problems at his job and he wants to quit, and she stays all day thinking negatively, that triggers her to smoke, she said that she has had depression in the past and sometimes she feels depressed again (she used to go to a psychiatrist but she had to have an interpreter and that was not comfortable for her), the doctor gave pills to her and she started to hallucinate and she refused to take them, she felt that this didn’t help her and she stopped going to the psychiatrist”*

(CHWRH Fair).

Participants with good CHWRH were not immune to feelings of depression and anxiety, but overall CHWs noted positive appraisals of emotional well-being among patients with good CHWRH. CHWs described participants as calm, positive, fulfilled, happy, and/or emotionally stable.

Related to emotional well-being, CHWs reported participants with poor CHWRH feeling less independent relative to individuals with good CHWRH. More specifically, CHWs described how uncontrolled chronic diseases, severe physical pain, or trauma led to a significant loss of mobility which prevented participants from carrying out daily activities and/or being able to care for themselves.

*“Participant has absence epilepsy, when she has a convulsion which lasts approximately one minute she says that when this happens she ends up on the floor, she says that her doctor recommends she can’t drive nor cook...she told me that the situation makes her feel desperate because she’s isolated all day and only goes to the store when her husband takes her...”*

(CHWRH Fair).

Additionally, CHWs recounted instances of participants’ familial stress, which was more common among patients with poor CHWRH. CHWs captured a wide range familial issues, which included interpersonal conflicts, death of a family member, family members with health problems, family separation (i.e., a family member living in Mexico), challenges with caring for children, marital conflict, and domestic violence.

*“Emotionally has problems, her son is in prison…”*

(CHWRH Fair).

Lastly, CHWs emphasized economic stressors primarily among patients with poor CHWRH. Financial challenges stemmed from difficulties in paying for medical care, lack of insurance, unemployment, being the sole earner within the home, and general problems paying for basic needs (rent, car payments, food). Because participants with poor CHWRH tended to have more complex health issues, CHWs also noted when financial struggles affected participants’ ability to manage their illness or access necessary medical treatment.

*“For a long time she has needed to see a urologist because she can’t control her urine, but she says she can’t pay for the appointment...she needs a lot of medical services but she unfortunately doesn’t have the money and she says there are many new ways in which she could reduce her pain but they would cost up to $15,000 and this is too much for her”*

(CHWRH Fair).

## 4. Discussion

The goals of the current study were to quantitatively examine the relationship between CHWRH and participants’ mental health outcomes and qualitatively assess what factors inform CHWs’ rating of health status. CHWRH showed a statistically significant association with depressive symptoms and emotional problems severity, which were above and beyond the contributions of the more heavily researched SRH. Qualitative findings demonstrate that CHWs appraise health in a holistic manner, incorporating elements of mental health they may be in a unique position to assess. Moreover, the qualitative findings highlighted the breadth of factors that were salient to CHWRH. CHWs not only drew from participants’ descriptions of their physical health, but also their ability to manage their disease, their emotional well-being, and social determinants of health, showing their comprehensive and ecological approach in understanding Latino patients’ health status. In integrating qualitative and quantitative findings, participants’ level of pain, ability to manage their health condition, emotional state, level of independence, familial stress, and economic stress seemed to contribute to CHWs’ rating of more or less favorable overall health.

While we contend the mixed-methods approach, relatively large sample and novel assessment of overall patient health from CHWs are strengths of this study, there are important limitations. First, the data are cross-sectional so the statistical models presented do not shed light on the long-term predictive ability of CHWRH. Additionally, as subjective raters, there is a potential for bias on the part of the CHWs in rating participants’ overall health status. Also, generalizability of the study results may be constrained. The three CHWs rating participant health were all relatively experienced and were part of a study that was intentionally focused on emotional health. Thus, the associations drawn between their rating of health and participants’ mental health outcomes may not be generalizable to the entire CHW workforce. It may be the case that because this study was focused on emotional health, the CHWs were particularly astute to participants’ mental health when appraising their health status. Furthermore, the unique cultural, geographic, and socioeconomic characteristics of the CHWs and study participants, who were primarily foreign-born Latino adults in economically and health care access challenged U.S.–Mexico border communities, may further limit the generalizability. In a different setting, patients may face different life and health challenges, which could change the factors CHWs take into account when appraising their health and ultimately the strength of the associations between CHWRH and mental health. More specifically, future research should examine if associations between CHWRH and mental health differ among foreign-born and U.S.-born Latino adults, perhaps due to stressors related to immigration status which can impact migrants’ mental health. However, it is important to reemphasize that, by definition, CHWs are from the communities served and are more likely to be sensitive to the contextual factors impacting their patients.

Limitations withstanding, our findings suggest that CHWs can effectively identify and engage Latinos in discussions regarding their mental health, and in these conversations are likely to be attuned to interconnections of patients’ depressive symptoms and their overall health. In their initial contact with study participants, CHWs ascertained rich information about participants’ daily lives and the circumstances that are or may be influencing their mental health. Considering challenges faced by Latino patients in receiving a mental health diagnosis and treatment within traditional primary care, CHWs could thus contribute to identifying patients in need of mental health services. More specifically, studies have shown that poor physician–patient communication contributes to lower rates of mental health treatment among Latinos [14,44,45]. A qualitative study assessed gaps in perceptions of mental health care between physicians and Latino migrant patients in one of the LINKS study sites, where participants expressed their desire to talk about their emotional well-being. Participants articulated that communication around mental health could be improved by their physicians initiating conversations about what is going on in their lives and how they are feeling [46]. Moreover, participants explained that life events directly influence their mental health, thus are important to discuss in assessing Latino patients’ mental health. However, physicians are often challenged by short appointment times and lack of cultural competence and/or language proficiency to successfully engage with Latino patients [47,48]. CHWs can address this critical challenge in health/mental health care, given that CHWs have demonstrated success in cultivating the trust and rapport necessary to facilitate conversations about patients’ lives and emotional well-being. Thus, our study suggests that embedding CHWs within health care systems (particularly those that serve a high proportion of Latino patients) could improve the identification of Latino patients with mental health needs and their subsequent mental health service utilization.

### Recommendations for Practice

As primary care and behavioral health care services become increasingly integrated, our findings underscore the potential contributions of the CHW workforce to transformative community approaches to promote the mental health of Latino adults with chronic disease. There is other evidence that CHWs are efficacious in numerous roles in the provision of mental health care [26,49]. First, CHWs can outreach to patients to connect them to health providers, in other words act as a “bridge” between community and health care services [12]. Community clinical linkages (CCLs) models are one ideal mechanism that could (1) position CHWs within systems of care and (2) leverage CHWs’ core competencies to improve patient health outcomes [50]. Within CCLs, clinical and community-based organizations collaborate to link patients to specific services to address external barriers to health care delivery [51]. Several studies have demonstrated that within CCLs, CHWs successfully act as linkage brokers connecting community residents to clinical resources based upon patients’ health needs [50]. Through CCLs, CHWs could interface with Latinos in clinical or community settings to assess their health, then identify individuals with unmet mental health needs, and connect them to appropriate care.

Patient follow up is also critical to the CCL model in order to ensure that patients not only access health services that they need and but also utilize them. A systematic review identified auxiliary support of mental health treatment (i.e., case management) as another important of role of CHWs in promoting patient adherence to mental health treatment [26]. Given that mental health disorders (depression, anxiety) are often associated with chronic disease, it is most likely that primary care physicians have the opportunity to diagnose mental health issues. Within primary care settings, CHWs can provide several areas of support that could improve Latinos’ utilization of mental health services. CHWs can work alongside physicians as part of patients’ health care teams within primary care in order to enhance communication between patients and providers [49]. The qualitative data highlighted the depth of information participants felt comfortable sharing with the CHW about their lives, which is important to physicians’ understanding of their patients’ mental health. Having CHWs work closely alongside physicians could ensure that doctors obtain comprehensive information about Latino patients’ emotional well-being and the specific challenges influencing their mental health.

## 5. Conclusions

The current study extends the current literature on SRH by examining CHWRH, which is novel to public health research. The results empirically suggest that CHWs rate patient health in a manner that is indicative of mental health status among Latino adults. The qualitative analysis demonstrates that from one interaction, CHWs gained meaningful insight into participants’ lives, and thus were able to rate health from holistic and ecological perspectives. Thus, the results provide empirical support for increased integration of CHWs in mental health care through approaches that directly situate CHWs within community-based mental health initiatives. The CHW workforce may be able to address deficiencies within primary care that impede the diagnosis and treatment of mental health issues among Latino communities through improved identification of Latino patients in need of mental health services. Thus, researchers should further study innovative models that establish a community-clinical continuum of care, such as CCLs, in order to address disparities in identification and treatment of mental health.

## Figures and Tables

**Table 1:** Baseline demographics, emotional well-being, self-rated health, and CHW-rated health of LINKS participants ^ab^.

Variable	N = 189
**Sex**, *n* (%)	
Male	27(14.3)
Female	162(85.7)
**Age**, mean (SD)	56.6(13.9)
**Ethnicity**	
Hispanic/Latino	179(94.7)
Not Hispanic/Latino	6(3.2)
Other/no answer	4(2.1)
**Education** (**years**)**,** mean (SD)	10.5(4.6)
**Nativity**, *n* (%)	
Born within U.S.	30(15.9)
Born outside of U.S.	159(84.1)
**Self-report chronic disease status**, *n* (%)	
Diabetes	71(38.6)
Asthma	17(9.2)
Cancer	12(6.5)
Hypertension	74(40.2)
Dyslipidemia	31(16.8)
Heart disease	26(14.1)
Chronic pain	37(20.1)
Vision problems	55(29.9)
**CES-D-10 score**, median (IQR)	7.0(5.0)
Missing, *n* (%)	17(9.0)
**Emotional problems severity**, *n* (%)	
Not at all	74(39.6)
Slightly	46(24.6)
Moderately	41(21.9)
Quite a lot	23(12.2)
Extremely	3(1.6)
**Self-rated Health**, *n* (%)	
Poor	17(9.0)
Fair	70(37.2)
Good	76(40.4)
Very good	19(10.1)
Excellent	6(3.2)
**CHW-rated Health**, *n* (%)	
Poor	9(4.8)
Fair	60(32.1)
Good	69(36.9)
Very good	43(23.0)
Excellent	6(3.2)

Abbreviations: CHW, Community health worker; LINKS, Linking Individual Needs to Community and Clinical Services; SD, standard deviation; CES-D-10, 10-item Center for Epidemiologic Studies Depression Scale Revised; IQR, interquartile range. ^a^ Some percentages do not sum to 100 due to rounding or missingness ^b^ Missing data rates were less than 5% unless otherwise specified.

**Table 2:** Multilevel linear models examining depression symptomology and emotional problems severity ^a^.

	Model 1: Depression Symptomology (*n* = 168) ^b^				Model 2: Emotional Problems Severity (*n* = 182) ^c^			
	β	SE	*p*	95% CI	β	SE	*p*	95% CI
Variable								
**Intercept**	6.85	2.14	0.002	2.61–11.09	1.92	0.28	0.002	1.36–2.48
**CHW-rated health**	2.52	0.57	<0.001	1.39–3.64	0.34	0.10	<0.001	0.15–0.54
**Self-rated health**	0.73	0.58	0.21	−0.43–1.88	0.20	0.10	0.21	−0.01–0.41
**Gender**								
Referent	--	--	--	--	--	--	--	--
Female	1.76	1.21	0.15	−0.63–4.15	0.47	0.22	0.15	0.03–0.90
**Education**	−0.19	0.10	0.07	−0.39–0.02	0.006	0.02	0.07	−0.03–0.04
**Nativity**								
Referent	--	--	--	--	--	--	--	--
Born outside of U.S.	−1.16	1.20	0.34	−3.54–1.21	−0.30	0.21	0.34	−0.72–0.11
**Age**	−0.06	0.03	0.09	−0.12–0.01	−0.01	0.01	0.09	−0.02–0.001

Abbreviations: CHW, community health worker; SE, standard error; CI, confidence interval. β Estimates are unstandardized beta coefficients. --Referent category. CHW-rated health and Self-rated health were coded 1–5, with higher scores indicating worse health status. ^a^ Multilevel linear models include CHW as the random factor. ^b^ Depression symptomology as measured by the CES-D-10, possible range 0–30. ^c^ Emotional problems severity as measured by one item of the SF-8, possible range 1–5.

**Table 3:** Emerging themes and sample quotes from the content analysis ^a^.

Theme	Description	Quotes
**Physical Health Issues**	Issues regarding participants’ physical and health conditions that increased their risk for disease.	“Participant is overweight and she states that she has prediabetes…”
Chronic	Having a chronic disease diagnosis.	“Participant has diabetes, arthritis, and high blood pressure, and some pain in his knees.”
Physical Pain	Struggles with physical pain, pain severity, and resulting disability.	“Participant has been dealing with chronic hand pain that limits daily activities.”
Acute	Traumatic or emergent health issues.	“Participant has worked all her life. She had a work accident almost two years ago and since then, she has not been able to work…”
Dental	Dental and oral health issues.	“She has also had a lot of problems with her teeth and because her medical insurance doesn’t cover dental she went to Mexico to be seen.”
**Emotional Well-Being**	Feelings participants were experiencing in their day to day lives, outlook on life, and/or a known mental health diagnosis.	“The participant has prediabetes and some depression since she lost her husband (2 years ago) and she is not making any effort to lower her sugar level.”
Resilience	Participants’ positivity in the face of adversity.	“The participant at this moment has several health problems however she takes care of herself and her sugar level and maintains a positive attitude.”
Independence	Feelings related to participants’ ability (or inability) to take care of their own needs.	“Participant had a [motor vehicle accident] 4 months ago. She has not been able to work since then. She describes herself as an independent woman and feels depressed because she is unable to do things herself and needs to ask for help. Emotionally she is very sad and worries that she won’t be able to go back to her normal life.”
**Self-Management and Care**	Participants’ effectiveness in managing their chronic disease and/or successfully accessing necessary medical care.	“Participant doesn’t take much care of her diabetes, she doesn’t measure her sugar level because she is afraid of [poking] herself, she is overweight and [does] not eat healthy, says that her doctors want that she gets a surgery to lose weight...”
**Readiness to Change**	Participants’ expressing willingness or desire to change their health behavior.	“Participant is overweight and she states that she has prediabetes, she doesn’t take good care of herself, and she wants to start making changes in her diet … she told me that one of her son has autism and he is overweight too, and she wants to do it for him too, sometimes she said is too hard to make these changes but she is sure she want to make it.”
**Family Stress**	Accounts of participants’ conflicts within their families.	“He is in charge of 6 kids here in USA and is worried if something happens to him, who will take care of them. His wife lives in San Luis, Mexico and he tries to go there with the children at least twice a week.”
**Economic Stressors**	Participants’ financial struggles.	“He is having financial problems. He is the only one that works because his kids are in school and he is in charge of them.”
**Social Support**	Participants’ perceptions of receiving or lacking care or support from family members or friends.	“Participant is recovering from a heart surgery she state she is doing great but she can’t exceed walking or doing things in her house, with her diabetes she told me that some days her sugar level is high…it has been very difficult for her lower her sugar level, and now she is almost blind of her left eye, but she is seeing a doctor who is helping her with her eye, she is in good hands she said and her neighbor help her almost the time (while her husband works).”

^a^ subthemes fall immediately under primary theme and are noted by non-bolded text.

## Data Availability

The data presented in this study are available on request from the corresponding author.
